# A Study on the Fault Location of Secondary Equipment in Smart Substation Based on the Graph Attention Network

**DOI:** 10.3390/s23239384

**Published:** 2023-11-24

**Authors:** Xian-Ming Xiang, Xiu-Cheng Dong, Jin-Qing He, Yong-Kang Zheng, Xin-Yang Li

**Affiliations:** 1 Sichuan University Jinjiang College, Meishan 620860, China; 212021085800048@stu.xhu.edu.cn (X.-M.X.); he.jq.26@foxmail.com (J.-Q.H.); adagioxf@stu.xhu.edu.cn (X.-Y.L.); 2 School of Electrical & Electronic Information, Xihua University, Chengdu 610039, China; 3 State Grid Sichuan Electric Power Research Institute, Chengdu 610099, China; zyk555@163.com

**Keywords:** smart substation, secondary equipment, fault diagnosis, graph attention network

## Abstract

The inability to locate device faults quickly and accurately has become prominent due to the large number of communication devices and the complex structure of secondary circuit networks in smart substations. Traditional methods are less efficient when diagnosing secondary equipment faults in smart substations, and deep learning methods have poor portability, high learning sample costs, and often require retraining a model. Therefore, a secondary equipment fault diagnosis method based on a graph attention network is proposed in this paper. All fault events are automatically represented as graph-structured data based on the K-nearest neighbors (KNNs) algorithm in terms of the feature information exhibited by the corresponding detection nodes when equipment faults occur. Then, a fault diagnosis model is established based on the graph attention network. Finally, partial intervals of a 220 kV intelligent substation are taken as an example to compare the fault localization effect of different methods. The results show that the method proposed in this paper has the advantages of higher localization accuracy, lower learning cost, and better robustness than the traditional machine learning and deep learning methods.

## 1. Introduction

The safety and reliability of secondary equipment can ensure the safe operation of primary equipment such as bus bars, circuit breakers, and main transformers. Once the secondary equipment is damaged or malfunctions, it affects the normal operation of the primary equipment and the secondary system [1,2,3].

Regular methods for locating faults in secondary equipment include the “empirical method” and “detection method”. The former is simple and convenient, and it is mainly used for locating simple faults. The latter is complex and is mostly used for locating difficult faults accurately. The secondary system contains a large number of information but lacks effective processing methods. The fault identification of secondary equipment mainly relies on the information assistance of the device and the work experience of staff members, which has low efficiency and accuracy of the diagnosis. Therefore, it is crucial for the construction and development of smart substations to propose a new method for the fault diagnosis of secondary equipment [4,5,6].

In the current fault diagnosis scheme, some researchers have proposed an improved fault tree method that adopts the structural entropy weighting method to assign different weights to every protection function after analyzing the connection between secondary equipment faults and alarm signals. Then, a fault diagnosis model is constructed. However, when there is a large number of complex alarm situations, the fault location result is inaccurate [7]. In [8], the authors construct a mapping relationship between the physical and virtual circuits of the secondary equipment by analyzing the SCD files. Then, the evidence table method and D–S criterion are combined to locate the malfunctioning equipment. However, it takes a lot of time to parse the key data.

In recent years, machine learning, neural networks, and other artificial intelligence technologies are gradually being used in smart grids [9]. Ren and Chen successfully applied a deep neural network and long short-term memory network (LSTM) to obtain a secondary equipment fault diagnosis. However, both methods only consider the fault diagnosis of a single interval. Currently, faults in secondary equipment usually involve multiple intervals. At the same time, the methods require a large number of samples, which results in high training costs [10,11,12]. Zhang proposed a method based on a graph neural network (GNN); however, the method is not suitable for dealing with dynamic graphs. When a new interval is added to a substation, its topology graph will change accordingly, and, thus, the approach will be no longer applicable. In addition, the model needs to be updated frequently when the amount of data changes [13].

In summary, the fault location of secondary equipment in smart substations has the following problems:

(1) The network structure of the secondary system for the smart substation is huge and complex and the information generated is also extremely complicated. However, there is a lack of effective methods to analyze and process it;
(2) Traditional diagnostic methods are inefficient and difficult to guarantee accuracy;
(3) Artificial intelligence methods have poor portability with high training costs and often require retraining the model.

In view of the shortcomings of the above methods, this study proposes a fault diagnosis method applicable to multiple intervals. The method has low training cost, high localization accuracy, and convenient model updating. By analyzing the information flow of the substation line intervals, the characteristic information exhibited by the corresponding online detection nodes when secondary equipment faults occur and the conventional methods of processing them are described. Furthermore, based on the online monitoring information of the secondary equipment, a representation method of fault feature information is proposed. Graph structure data are automatically constructed from the extracted feature information according to the K-nearest neighbor (KNN) algorithm. Based on the graph attention network (GAT), a fault localization model is built that takes the data in the form of a graph structure as an input to obtain the output of specific fault points.

## 2. Fault Data Detection and Characterization of Fault Signature Information

Smart substations adopt IEC 61850 standard [14] communication protocols and data models, which can realize connection and data exchange between devices. The “three layers and two networks” system, respectively, consists of the process layer, interval layer, station control layer, the process layer network, and station control layer network in the substation. The system realizes the real-time monitoring, remote control, and fault diagnosis of the substation equipment and improves the operation efficiency and reliability of the substation [15]. The IEC61850 protocol is used from the process layer to the control center for information interaction in the substation. If abnormalities and faults occur in the main equipment in the substation, the system will protect the main protection according to the parameters already set, record the situation in the period, and provide a status analysis report [16].

### 2.1. Fault Data Detection

**Figure 1:**
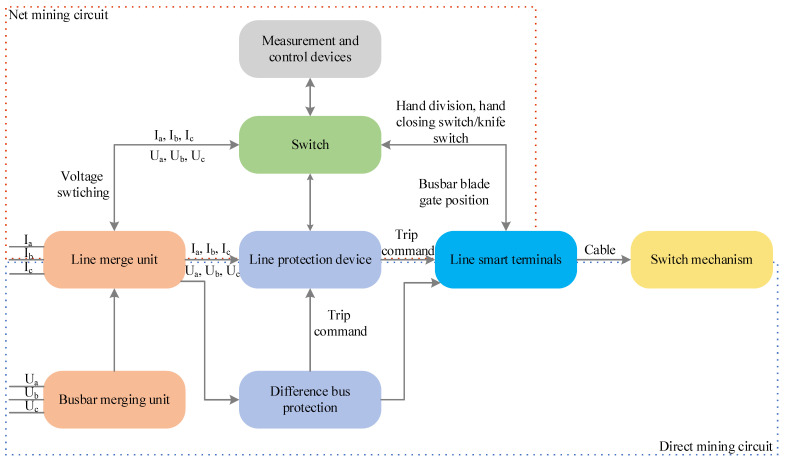
Line protection information flow.

The fault diagnosis objects of the IEC61850-based intelligent substation process layer mainly include devices and communication links between devices. The process layer devices mainly consist of merging units, intelligent terminals, protective devices, measurements, control devices, etc. The intelligent substation online detection system can collect the various parameter data of the substation in real time and analyze and process them in a certain way [17,18]. When a fault occurs, the redundant detection of secondary devices by arranging detection nodes (e.g., message reception status of secondary devices, alarm messages issued by devices, traffic size of messages, etc.) is the data basis for effectively identifying the location of the fault. Taking the line protection unit in Figure 1 as an example, when it fails, the line merging unit will send its collected voltage and current signals in the form of sampled value (SV) messages to the protection unit, which will then send a Generic Object-Oriented Substation Event (GOOSE) trip message to the line intelligent terminal, which will finally isolate the faulty equipment or line. At this time, the detection system collects the self-test information, message information, and other information: the normal sampling of the measurement and control device, the alarm of the protection device’s sampling interruption, and the collection method are also directly collected. Finally, the SV channel fault can be obtained through the existing fault reasoning knowledge base and its experience after technicians receive the relevant detection information.

As for some common faults, the technicians can locate them through detected information and a fault reasoning knowledge base. The relevant detection system only aims to collect and analyze the messages and alarms in the network; however, the final fault location and analysis of the faults need to be completed by the operation and maintenance staff. Moreover, in the face of huge and complicated data information, it is very difficult to rely on the experience of operation and maintenance personnel to accurately locate the fault [19].

### 2.2. Characterization of Fault Signature Information

Facing the large amount of information data generated by the secondary system, according to the characteristics of different secondary devices in substations and the need for online monitoring and the fault diagnosis of secondary circuits, the main information to be monitored should include (1) the operating status information of devices; (2) alarm information; (3) communication message traffic status information; (4) SV/GOOSE operating status information [20,21].

In this study, the collected monitoring information is integrated into the specific case as shown in Equation (1):

(1) $$V_{x}=\left\{X_{A},X_{B},X_{C}\right\}$$

In (1), $V_{x}$ represents the feature information set of the Xth node (the Xth event), where $X_{A}$ integrates the self-test status information of the merging unit, line protection, and intelligent terminal, as shown in Equation (2):

(2) $$X_{A}=\left\{X_{M1},\cdots ,X_{Mi},X_{P1},\cdots ,X_{Pj},X_{IT1},\cdots ,X_{ITk}\right\}$$

In (2), $X_{Mi}$, $X_{Pj}$, and $X_{ITk}$ represent the status information of the merging unit, protection device, and intelligent terminal, respectively, as shown in (3):

(3) $$\left\{ \begin{array}{l} X_{Mi}=\left\{X_{Mi\_T},X_{Mi\_S},\cdots ,X_{Mi\_A}\right\}\\ X_{Pi}=\left\{X_{Pi\_T},X_{Pi\_S},\cdots ,X_{Pi\_A}\right\}\\ X_{ITi}=\left\{X_{ITi\_T},X_{ITi\_S},\cdots ,X_{ITi\_A}\right\} \end{array}\right.$$

In (3), the corresponding self-test abnormal information $X_{Mi\_T}$ synchronization abnormal information $X_{Mi\_S}$ and device lockout status $X_{Mi\_A}$ and other characteristic information are recorded in the case of $X_{Mi}$.

$X_{B}$ in (1) represents the secondary system of measurement and control devices, intelligent terminals, line protection, bus protection, and other related secondary equipment message acceptance status information, as shown in (4):

(4) $$\left\{ \begin{array}{l} X_{B}=\left\{message_{1},message_{2},\cdots ,message_{k}\right\}\\ message_{k}=\left\{message_{k1},message_{k2},\cdots ,message_{km}\right\} \end{array}\right.$$

Each $message_{k}$ in (4) represents the set of the accepted states of the kth message, and $message_{km}$ is the state information of the mth device subscribed to the message to receive the message, which is recorded as 1 if the message is accepted and 0 otherwise.

$X_{C}$ in (1) represents the collected three-phase voltage and current values, with a total of 12 sampled values.

Finally, all fault events are constructed into the form of graph structure data G=(V,E), where V represents the set of the resulting data samples, i.e., all nodes of the graph data in which the feature information of each sample is shown in $V_{x}$ above; E carries the relationship between the edges of the data samples, i.e., the adjacency matrix.

## 3. Graph Neural Network

A graph neural network (GNN) is a framework that has emerged in recent years to learn directly from graph-structured data using deep learning, and its excellent performance has attracted a high degree of attention and in-depth exploration by scholars [22,23,24,25]. Fault location in smart substation secondary equipment can be viewed as a classification problem, i.e., classifying nodes composed of different events and, thus, achieving fault location.

### 3.1. Graph

**Figure 2:**
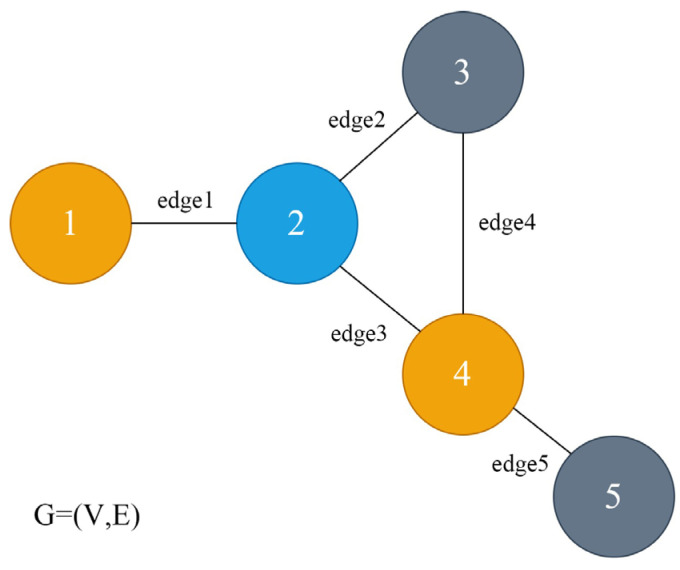
Graph structure.

A data structure consisting of nodes and edges between nodes is called a graph, as shown in Figure 2. A graph is expressed in the form of G(V,E), where G denotes a specific graph, V is the set of nodes in the graph G, each node has different feature information, the relationship between nodes is represented by edges, and E is the set of all edges in the graph G. E is the empty set.

### 3.2. Graph Neural Network

A GNN is a neural network that acts directly on the graph structure and processes data according to the node characteristics and structural features of the graph, and its information is propagated as shown in Equations (5) and (6).

(5) $$h_{i}=f(x_{i,node},x_{i,edge},h_{i,neighber},x_{i,neighber})$$

(6) $$o_{i}=g(h_{i},x_{i,node})$$

In (5), $x_{i,node}$, $x_{i,edge}$, $h_{i,neighber}$, and $x_{i,neighber}$ represent the vertex feature information, edge feature information, neighbor node state information, and neighbor feature information of node i. In (6), f and g are the activation functions. $h_{i}$ and $o_{i}$ are the state information and output results of node i, respectively.

GNNs use the node feature information obtained from learning updates in the above way and edge feature information to perform tasks such as node classification, edge prediction, or graph classification. Among graph neural networks, the graph convolutional neural network (GCN) and graph attention neural network (GAT) are the two most widely used graph neural network techniques.

### 3.3. Graph Convolutional Neural Network

The graph convolutional neural network (GCN) is the pioneer of graph neural networks. Compared with GNN, GCN has a different way of information aggregation, and its information propagation between layers is shown in (7).

(7) $$h^{\left(n+1\right)}=\sigma ({\tilde{D}}^{-\frac{1}{2}}\tilde{A}{\tilde{D}}^{-\frac{1}{2}}h^{\left(n\right)}W^{\left(n\right)})$$

In (7), A is an N×N-dimensional adjacency matrix formed between N nodes, and $\tilde{A}$ is obtained from the matrix addition of matrix A with the unit matrix. $\tilde{D}$ is the degree matrix of $\tilde{A}$. $h^{\left(n\right)}$ is the input feature information of the Nth layer. $W^{\left(n\right)}$ is the parameter matrix. σ is the activation function. $h^{\left(n+1\right)}$ is the output information.

### 3.4. Graph Attention Network

**Figure 3:**
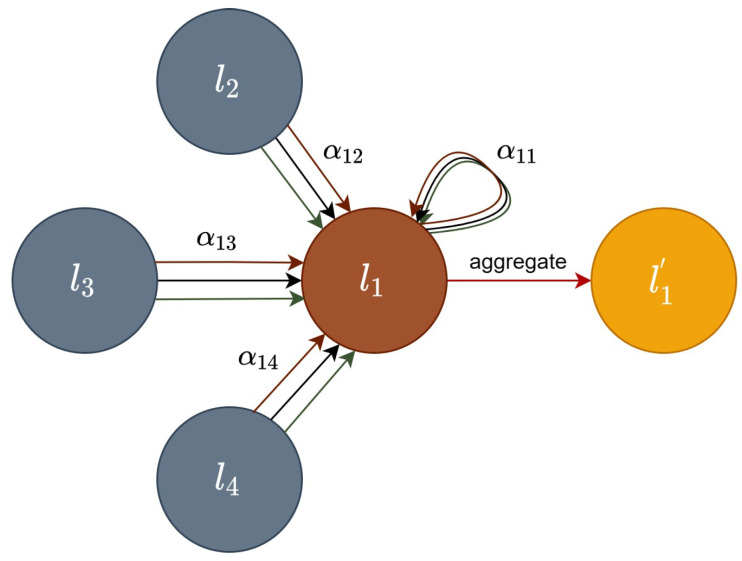
Node update mechanism.

The graph attention network (GAT) adds a hidden self-attentive layer to the GCN and assigns different weights to different nodes in the neighborhood in the convolution process by superimposing the self-attentive layer, and its node information update mechanism is shown in Figure 3.

First, node n calculates the similarity coefficient $e_{nm}$ between itself and its neighboring nodes, as shown in (8).

(8) $$e_{nm}=\alpha \left(\left[Wl_{n}∥Wl_{m}\right]\right)$$

In (8), $l_{n}$ and $l_{m}$ are the feature information of node n and its neighboring node m, respectively; W is the parameter matrix, and α is a mapping function, where the features obtained by splicing node n with m are mapped to a real number.

Then, the SoftMax function is used with the correlation coefficient $e_{nm}$ obtained above to calculate the attention coefficient $\eta_{nm}$ as shown in (9).

(9) ηnm=exp(LeakyReLU(enm))∑m∈Nexp(LeakyReLU(enm))

where N represents all neighbors of node n on the graph, and LeakyReLU is the activation function, which serves to prevent the loss of the feature information of node n after normalization.

Finally, the new feature information $l_{n}^{\prime }$ is obtained by the activation function σ after weighting and summing the features using the attention coefficients $\eta_{nm}$ obtained above, as shown in (10).

(10) $$l_{n}^{\prime }=\sigma \left(\sum_{}^{}{}_{m\in N}\eta_{nm}Wl_{m}\right)$$

**Figure 4:**
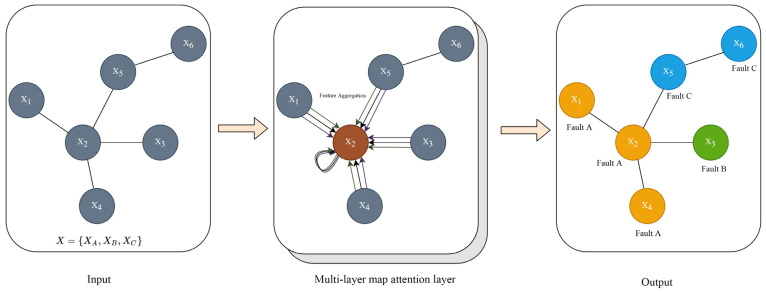
GAT model structure.

Each color in Figure 3 represents a different way of updating information, and repeating the above information’s updating process can obtain several different attentional features, and all the different features are aggregated into one overall feature to achieve the fitting effect. The structural model of GAT is shown in Figure 4. Both GCN and GAT networks learn new feature expressions by re-aggregating the feature information of the central node and its neighboring nodes to the central node, except that the former uses the Laplace matrix while the latter uses attention coefficients. Because of its different operation mechanisms, the GAT network is a good solution to the problem that the GCN network is not suitable for handling dynamic graphs, and it is more adaptable in the face of new data [26].

## 4. GAT-Based Secondary Equipment Fault Diagnosis Model Construction

### 4.1. K-Nearest Neighbor Algorithm

K-nearest neighbor (KNN) is a supervised learning algorithm that selects the K training samples that are closest to the input samples in the feature space and obtains the output according to the decision rules. The algorithm is simple, theoretically mature, and commonly used for classification and regression tasks. Among them, the selection of K values, the measure of distance, and the decision rule are the three basic elements of KNN. The computational procedure is shown below:

(1) Calculate the distances between the points to be classified and the known points and sort them in increasing order of distance;
(2) Select the K points with the smallest distance from the unknown points;
(3) Determine the number of occurrences of the category in which the first K points are located;
(4) Return the category with the highest number of occurrences of the first K points as the category of the unknown points.

### 4.2. Construction of the Graph Structure

The topological graph is an important cornerstone of the graph neural network, which can be constructed in various ways. The better the graph structure is constructed, the better it can reflect the relationship between network structures. The richer the extracted features are, the better the information is reflected [27,28]. Zhang extracted information from SCD files and stored it in the neo4j graph database in the form of nodes and edges. However, there are many ways to connect devices. Every time fault information appears, the network structure must be considered and the network configuration must be changed to update the secondary loop. This method requires a lot of effort to form the graph data structure in terms of the network topology diagram. When a new interval is added, the entire graph structure needs to be reconstructed. In this study, we focus on collecting important feature information after each fault event occurs. We regard each event as a node to learn the implicit connection and difference between them and achieve a fault diagnosis through the graph neural network. Therefore, the graph structure of this study finds the connection between nodes automatically based on algorithms. According to the information set of secondary equipment fault features obtained earlier, each data sample is regarded as a node in the graph, and, then, the KNN algorithm is used to assess the relationship between nodes, as shown in (11).

(11) $$\left\{ \begin{array}{l} Edge_{mn}=KNN\left(k,D_{mn},d_{m}\right)\\ D_{mn}={\left(\sum_{q=1}^{p}{\left(l_{m}^{\left(q\right)}-l_{n}^{\left(q\right)}\right)}^{2}\right)}^{\frac{1}{2}} \end{array}\right.$$

In (11), k is the hyperparameter of the KNN algorithm. $D_{mn}$ is the distance metric formula used in the KNN algorithm (Euclidean distance is used in this study), which represents the distance relationship between node m and node n. $d_{m}$ is the set of distances between node m and the whole sample nodes. $l_{m}^{\left(q\right)}$ and $l_{n}^{\left(q\right)}$ represent the qth dimensional feature values of nodes m and n, respectively, and the total feature dimension of each node is p. When $D_{mn}$ is the k smallest value in $d_{m}$, $Edge_{mn}=1$, which means there is an edge relationship between node m and node n. Otherwise, $Edge_{mn}=0$, and there is no edge relationship between the two.

Through the above, we can obtain the adjacency matrix A between the nodes and then add 1 to the diagonal of the adjacency matrix to obtain $\tilde{A}$ and turn it into a self-loop graph. Thus, we obtain the whole graph data structure, or “Graph”.

### 4.3. Fault Data Sample Expansion

In order to realize the autonomous training of deep learning fault diagnosis models, a large set of fault samples needs to be provided. A common practice is to obtain data through the accumulation of previous fault events in substations; however, this model requires a certain accumulation time. In addition, the high reliability of certain equipment during actual operation leads to a lack of samples when this type of equipment fails. In addition, some of the samples also have missing alarm information. In short, the existing fault data actually obtained from smart substations have problems such as insufficient sample size and uneven sample distribution. Therefore, in order to better help train the model, it is necessary to generate other reliable samples in addition to utilizing the existing dataset.

**Figure 5:**
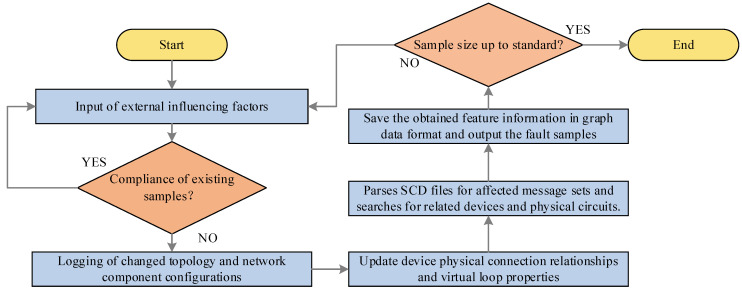
Fault data expansion process.

First, the range of faults involved is determined from the available fault data. Considering the entire fault range as a system whole, external influences (e.g., different component failures, changes in network topology diagrams, network component configurations, etc.) are fed into the system beforehand. Then, the physical and logical connections between devices as well as the relationship between message transmission and subscription are obtained by parsing the smart substation SCD file. Due to the influence of external factors, the switches, ports, fibers, etc., in the original network system produce new operating states. The new state information of each device node is collected separately and the obtained data are stored in the form of graph data as described in the previous section. The specific flow is shown in Figure 5.

### 4.4. Data Preprocessing

In order to make the original data more suitable for neural network training and improve its training effect, this study first uses the PCA dimensionality reduction method to reduce the original data and then uses the Min–Max method to normalize the reduced data. The PCA dimensionality reduction steps are as follows:

(1) Form the data into an m×n-dimensional matrix Y. The covariance matrix is found by subtracting the mean of each row of Y from the mean of the changed row;
(2) Find the eigenvalues of the covariance matrix and the corresponding eigenvectors and arrange the eigenvectors into a matrix from top to bottom according to the corresponding eigenvalue magnitude; then, take the first i rows to form the matrix G;
(3) y = GY is the data obtained after dimensionality reduction.

The data obtained above are Min–Max normalized, and the Min–Max method is shown in (12).

(12) $$X_{m}^{\prime }=\frac{X_{m}-X_{\min}}{X_{\max}-X_{\min}}$$

In (11), $X_{m}$ represents any value among all data in the data, and $X_{\max}$ and $X_{\min}$ represent the maximum and minimum values in the dataset, respectively. $X_{m}^{\prime }$ is the final value obtained, and its value range is (0,1).

### 4.5. GAT Diagnostic Model

**Figure 6:**
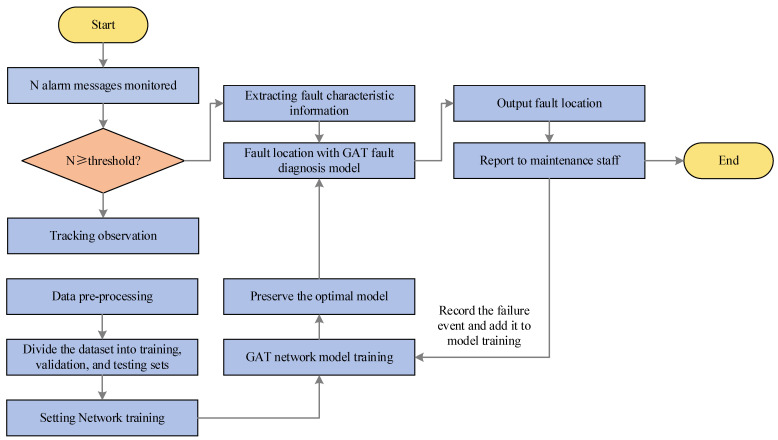
Fault location framework.

With the problems of the secondary equipment fault location being regarded as a GAT node classification task [23], the collected data are divided into two parts according to the graph structure model built earlier. One is input into the network for training and the other is used to test the performance of the network model. The fault localization framework is shown in Figure 6, and the specific steps are shown below.

(1) A diagnostic model is trained. Firstly, the obtained data are preprocessed and then divided into a training set, validation set, and test set. Finally, the dataset is fed into the GAT network to train and save the optimal model;
(2) When a fault message is detected, the total number of its feature messages is first counted and recorded as N. Whether N is greater than a threshold value that is the minimum number of feature messages collected when a previous fault occurs must be determined. If N is greater than the threshold value, the next step of diagnosis is performed; otherwise, the tracking observation continues;
(3) The extracted fault feature information is constructed as the feature information expression described in the previous section and is input into the already trained GAT diagnostic model for fault location;
(4) The diagnosis results are output and the current diagnosis results are added to the old fault database for model training.

### 4.6. Model Evaluation Criteria

The evaluation metrics used in this study for the model are F1-Score and Accuracy. The F1-Score metric measures the overall performance of the different models, and Accuracy focuses on the positioning accuracy of the model. Its specific explanation is shown below:

TP: positive samples are correctly predicted as positive samples; FP: negative samples are incorrectly predicted as positive samples; TN: negative samples are correctly predicted as negative samples; FN: positive samples are incorrectly predicted as negative samples.

Aaccuracy consists of TP, FP, TN, and FN, as shown in Equation (13):

(13) $$Aaccuracy=\frac{TP+TN}{TP+TN+FP+FN}$$

The F-score consists of precision and recall rates, as shown below:

(14) $$\{\begin{array}{l} Precison=\frac{TP}{TP+FN}\\ Rrecall=\frac{TP}{TP+FN}\\ F-Score=(1+\beta^{2})\frac{Precison\cdot Rrecall}{\beta^{2}\cdot Precison+Rrecall} \end{array}$$

In Equation (14), the meaning of β is shown in (15):

(15) $$\{\begin{array}{l} \beta =1,\mathrm{Accuracy}\;\mathrm{and}\;\mathrm{Recall}\;\mathrm{are}\;\mathrm{equally}\;\mathrm{important}.\\ \beta <1,\mathrm{Accuracy}\;\mathrm{is}\;\mathrm{more}\;\mathrm{important}.\\ \beta >1,\mathrm{Recall}\;\mathrm{is}\;\mathrm{more}\;\mathrm{important}. \end{array}$$

The F-Score is the F1-Score when β=1, and the F1-Score is used as the evaluation index.

## 5. Case Analysis

### 5.1. Case Introduction

**Figure 7:**
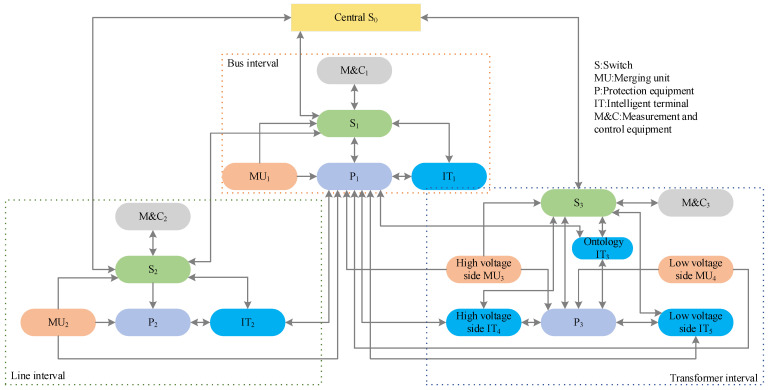
Interval topology diagram.

In this paper, a 220 kv smart substation is taken as an example, and its fault range includes one bus interval, one line interval, and one transformer interval. The expanded dataset is obtained as shown in Section 3 and Section 4, and the example is used to test the effectiveness of the proposed method. The topology diagram of intervals mentioned above is shown in Figure 7. Table 1 shows the specific devices that are presented in Figure 7. $S_{0}$ in the figure represents the center switch, and $S_{1}-S_{3}$ are the switches of each interval. Table 2 shows the information flow of the interval, which records the subscription relationship of messages among the devices. These subscription relationships are obtained by parsing the smart substation SCD files. In order to achieve the effect of accurate fault location, the faults are first classified into device and component faults themselves, as well as power supply faults, fiber and optical port faults between devices, inter-device connection faults, device configuration errors, communication network faults, etc. In total, there are 26 cases, as shown in Table 3. The expanded dataset is used as a sample for model training and testing. The collected 2080 data samples are labeled according to the above fault types and constructed into the structural form of graph data based on the method described in the previous section. With the abnormal sample of the line merging unit voltage taken as an example, the characteristic information is the protection unit alarm, total merging unit alarm, merging unit receiving bus, merging unit SV interruption, normal current value of line protection unit sampling with the zero value of voltageand normal voltage and current values of bus protection, etc. The information is constructed as an expression of Equation (1) and labeled with the corresponding fault label as a node in G=(V,E). Finally, 70% of the total data samples are used as the training set, 10% as the validation set, and 20% as the test set.

### 5.2. Effect of Different Hyperparameters on the GAT Model

**Figure 8:**
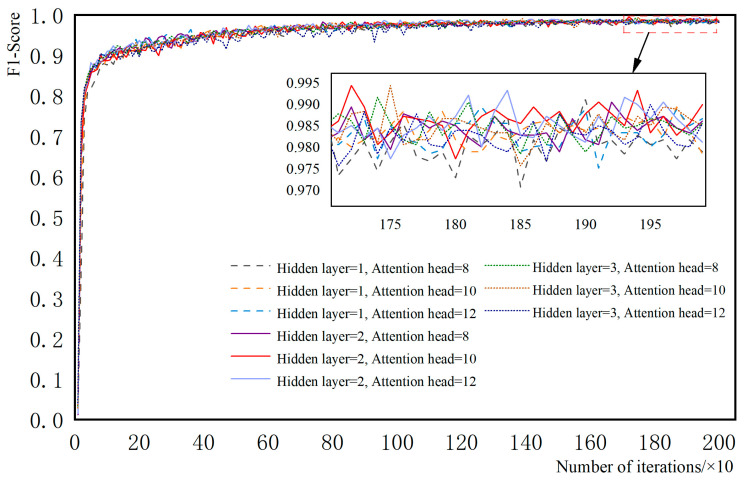
Effects of GAT layer and attention head on neural network optimization.

The appropriate selection of hyperparameters plays a crucial role in the final diagnostic performance of the model. The data were substituted into the model several times and the hyperparameters that mainly affect the GAT network were found to be the number of hidden layers of the network and the number of multi-headed attention heads, as shown in Figure 8. The other hyperparameters were selected as shown in Table 4 below.

In Figure 8, the horizontal axis is the number of iterations, and the numerical axis is the evaluation index. Here, the evaluation index is an F1 score value (it is the summed average of the precision and recall rates, with a maximum of 1 and a minimum of 0), and its larger value represents the higher quality of the model. From the final convergence in the figure, as the number of hidden layers and the number of attention heads increase, the F1 value becomes larger. When the number of hidden layers is 1 and the number of attention heads is changed, the F1 value changes significantly; when the number of hidden layers is 2, the value does not change much; when the number of hidden layers reaches 3, the value starts to decrease slightly. The F1 value of the training model alone cannot fully evaluate its final performance, and the generalization ability of the model should also be considered, with F1 being too high but not causing overfitting. The effect of the model in the test set with different hyperparameters is shown in Table 5.

From Table 5, it can be seen that the accuracy of the diagnostic results shows an increasing and then decreasing trend as the number of cryptic layers and the number of multi-attention heads increase while keeping other hyperparameters constant. Finally, the model works best when the number of layers is 2 and the number of attention heads is 10. Too many layers and heads will only increase the training time and cause overfitting.

### 5.3. Specific Case Analysis

(1) In this paper, an actual line merging unit sampling fault is used as an example. When it occurs, its associated measurement and control devices and protection devices are affected. The measurement and control device do not collect the corresponding voltage and current information, and the monitoring of the background telemetry data are also affected by the measurement and control device. The protection device collects the wrong voltage and current values, and, thus, the protection function is affected. The merging unit then sends out an abnormal device alarm, SV total alarm, and abnormal sampling alarm; the protection device also sends out an SV total alarm due to receiving wrong sampling information from the merging unit, and the protection device is blocked. The collected feature information is represented in detail in Equation (16).

(16) $$\{\begin{array}{l} \{\begin{array}{l} X_{A}=\left\{X_{Mi},X_{Pi},X_{ITi}\right\}\\ X_{Mi}=\left\{0,1,0,\ldots ,1,\ldots ,1,0,\ldots \right\}\\ X_{Pi}=\left\{1,0,\ldots ,1,1\right\}\\ X_{ITi}=\left\{0,0,\ldots ,0\right\} \end{array}\\ X_{B}=\left\{0,0,\ldots ,0\right\}\\ \begin{array}{l} X_{C}=\{3.012,3.026,3.033,\\ \;57.481,57.556,57.549,\\ \;2.966,2.956,2.884,\\ \;57.406,58.228,56.477\} \end{array} \end{array}$$

The non-zero values are mainly listed in Equation (16). All the above values are preprocessed and added to Equation $V_{x}{}^{\prime }=\left\{X_{A},X_{B},X_{C}\right\}$. The feature information is then input into the GAT network model to obtain the fault number, and, finally, the corresponding fault type is found in the fault table. The fault number obtained is number 1 (line merging unit sampling anomaly). Compared with the traditional diagnostic method proposed in [29], this study makes a comprehensive assessment of the operating status of the equipment by comparing the double AD sampling value of the protection device with the SV sampling value of the network message analysis device by analyzing the relevant information. When there is a line merging unit sampling fault, the double AD sampling value of the protection device and the SV sampling value of the network message analysis device will be abnormal. In this case, a fault diagnosis according to the method used in [24] yields incorrect results. The main misjudgments resulted in fault number 10 (line protection device—smart terminal’s smart terminal I/O board fault) and fault number 15 (line protection CPU fault).

(2) Take a protection device input port failure between the merging unit of the main substation interval and the protection device of the bus interval as an example. The protection device issues a self-test alarm due to abnormal operation. At the same time, the protection device issues an abnormal SV sampling data alarm due to inconsistent information received from the direct/net collection. The protection device issues a total SV alarm due to receiving wrong sampling information from the merging unit. The protection device issues a sampling interruption. The protection unit issues an alarm for sampling interruption and the protection unit locks out. The above messages are represented in the feature set $X_{A}$. Since there is no message loss, the elements in the feature set $X_{B}$ are all 0. The details are shown in (17):

(17) $$\{\begin{array}{l} \{\begin{array}{l} X_{A}=\left\{X_{Mi},X_{Pi},X_{ITi}\right\}\\ X_{Mi}=\left\{0,0,\ldots ,0\right\}\\ X_{Pi}=\left\{0,\ldots ,1,1,1,1,\ldots ,1,0,\ldots \right\}\\ X_{ITi}=\left\{0,0,\ldots ,0\right\} \end{array}\\ X_{B}=\left\{0,0,\ldots ,0\right\}\\ \begin{array}{l} X_{C}=\{0,2.9664,0,\\ \;57.581,6.2588,57.447,\\ \;0,2.9664,0,\\ \;57.5881,6.2588,57.447\} \end{array} \end{array}$$

The results obtained by substituting them into the GAT network and BP network are shown in (18) and (19), respectively:

(18) $$O_{\mathrm{GAT}}=\left[\begin{array}{cccccc} \mathrm{NO}.1 & \ldots & \mathrm{NO}.18 & \ldots & \mathrm{NO}.20 & \ldots \\ 0 & \ldots & 0 & \ldots & 1 & \ldots \end{array}\right]$$

(19) $$O_{\mathrm{BP}}=\left[\begin{array}{cccccc} \mathrm{NO}.1 & \ldots & \mathrm{NO}.18 & \ldots & \mathrm{NO}.20 & \ldots \\ 0 & \ldots & 1 & \ldots & 1 & \ldots \end{array}\right]$$

From the above results, we can see that the GAT model can make an accurate judgment for fault No. 20 (input port fault of the protection device of the combined unit-bus interval of the main transformer interval), while the BP model misjudges it as fault No. 18 (SV board fault of the protection device).

### 5.4. Comparison of Different Methods

**Figure 9:**
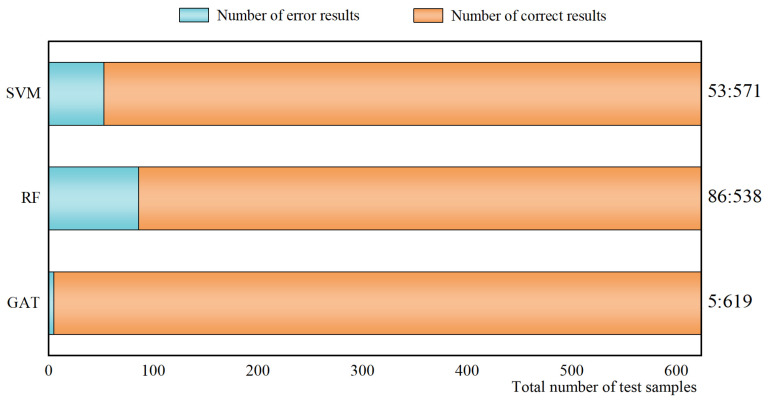
Discriminatory status of different methods.

(1) The method proposed in this paper is compared with the support vector machine (SVM) and random forest algorithm (RF) commonly used in machine learning. From 2080 total samples, 70% of them are selected as the training set, and the remaining 30% are used to test the effect of the fault localization of each method. The specific test results are shown in Figure 9. In order to show the results of the output, accuracy is used here as an indicator.

In Figure 9, there is a total of 624 test samples. From the final discrimination, SVM, RF, and GAT all have good localization effects. Meanwhile, it can also be seen that GAT has a stronger learning ability than SVM and RF, with fewer discrimination errors and higher localization accuracy. The specific discriminations of the three methods are shown in Table 6.

Table 6 shows in detail the specific discriminations of the different methods. The table mainly presents the faults that were misjudged, and the number of those that were not misjudged is indicated by “other”. From the table, it can be seen that more than half of the fault types can be accurately located by both machine learning and neural network algorithms. For some more complex fault types, such as the 24th fault in the table (failure of fiber optic link breakage of main transformer interval combining unit—bus bar protection device), the error rate of the diagnosis result is relatively higher. Because this type of fault often causes the equipment associated with this interval and other intervals to issue some characteristic alarms. The alarm signal often covers multiple devices in the interval, thus leading to the increased complexity of the collected characteristic information, which increases the difficulty in discrimination.

**Figure 10:**
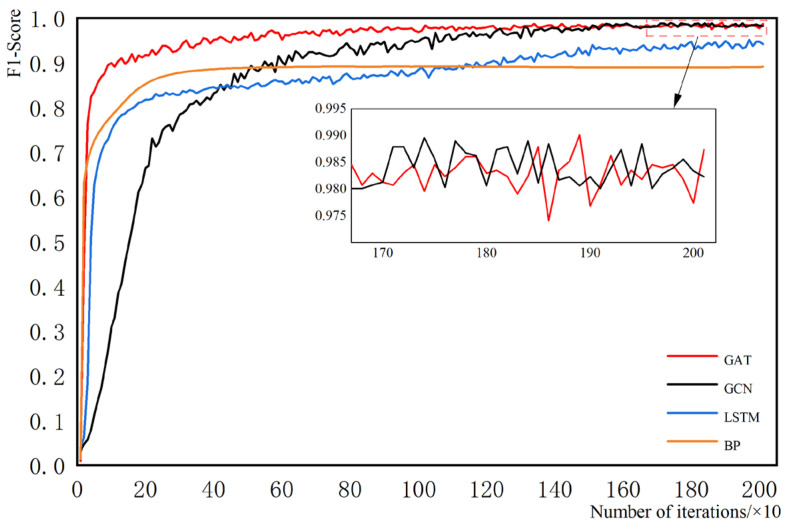
Comparison of different network models.

(2) The GAT model is compared with the traditional BP network, the LSTM network, and the GCN model, which is also a graph neural network. All of the models use the same dataset. The main hyperparameters are kept the same, such as the number of hidden layers is all 2, the number of hidden layer neurons is (16,32), the number of iterations is all 2000, etc. The evaluation metrics use the F1 score values, and their details are shown in Figure 10.

The time taken for 2000 iterations of each method is T1, and the time taken for each method to reach convergence is T2. The details are shown in Table 7.

From the comprehensive analysis of Figure 10 and Table 7, the convergence speed of the network, in order from fast to slow, is BP, GAT, GCN, and LSTM. Compared with the BP network, LSTM has a more complex network structure and gating mechanism. Its computational volume is bigger and the network runs slower, but it processes the data better, and the model performance index is higher. Unlike BP and LSTM, GCN and GAT are graph neural networks. The higher the number of nodes and edges in the graph, the more computationally intensive the graph neural network is. The results under the experimental conditions in this paper show that, compared to LSTM, GCN, and GAT have less computation, faster convergence, and better final localization. Compared to GCN, GAT reduces the amount of computation due to the introduction of the attention mechanism. From the results, GAT actually runs slower than GCN under the condition that the number of attentional heads is 10 (an increase in the number of attentional heads increases the computation of the network). However, due to the increase in the number of attentional heads, GAT has a better learning ability and the network reaches stabilization earlier than the GCN network.

In summary, by comparing the method proposed in this paper with commonly used machine learning methods (SVM, RF) and neural network methods (BP, LSTM, GCN), the following conclusions can be tentatively drawn:

(1) Compared with SVM and RF, GAT has a stronger learning ability and higher localization accuracy;
(2) Compared to BP, LSTM, and GCN, the GAT network has a faster training speed, higher performance metrics, and a better model fit.

## 6. Impact on the Model When the Graph Structure of the Training Set Does Not Match the Graph Structure of the Test Set

**Figure 11:**
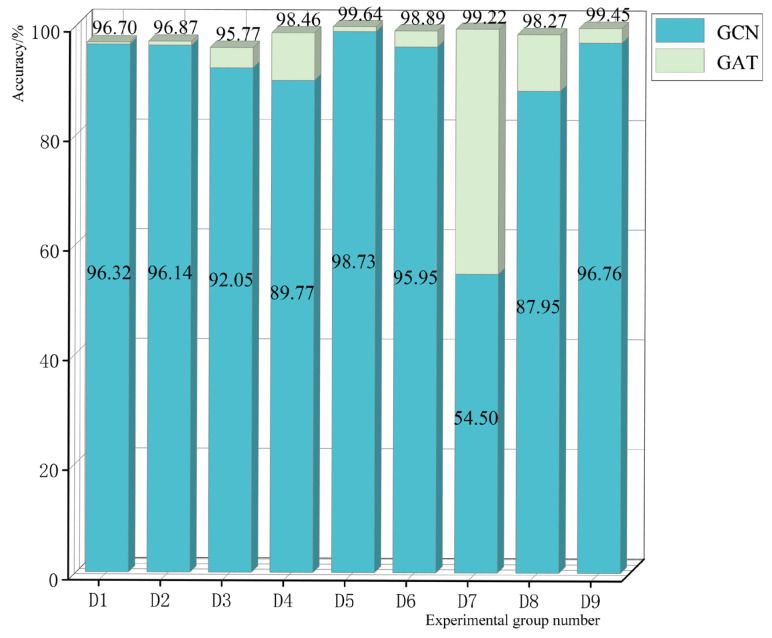
Test results for different graph structure datasets.

Graph data structures are constructed based on the KNN algorithm. In this algorithm, the value of K is a hyperparameter and the generated graphs are different when different values are set. To discuss the effect on model performance when the graph structures of the training set and test set are different, a comparison test was set up as shown in Table 8. The specific tests of comparison groups A, B, and C are shown in Figure 11.

As can be seen from Figure 11, when the K values of the training set and the test set are different, their effects on the model performance also differ. When there is a change in the K value, the accuracy of the GCN model decreases significantly, especially when the K value of the test set is smaller than the K value of the training set. In contrast, the results of the GAT model are almost unchanged. This is because the GCN model relies on the information of its entire graph structure when it is trained. If its graph structure is changed, the weight parameters trained will no longer be applicable. In contrast, the GAT model is trained with a linearly transformed parameter matrix of neighboring features, which is the same for any of its neighbors. After all, no matter which graph construction method is adopted, the graph structure of the test data may not always match the graph structure of the trained model. When a new interval is added to the substation, or when a new fault is added to the test set, the adjacency matrix of its graph data changes accordingly, and the prediction effect of the GCN model is significantly reduced at this time; however, the GAT model can better adapt to this new situation as it handles dynamic graphs better than GCN models.

## 7. Learning Sample Cost Comparison

For different substations, their specific conditions are different. The space for storing data in network loggers is limited, and their previous fault datasets are often incomplete and have an insufficient sample size. However, fault location models built on the basis of deep learning algorithms often require a large number of training samples, which contradicts the reality of insufficient data samples in smart substations. In this paper, model training has been aided by data augmentation methods. But practical situations can vary. The proposed method was compared with other common algorithms in order to verify the advantages of the algorithm used in this paper in terms of training cost.

**Figure 12:**
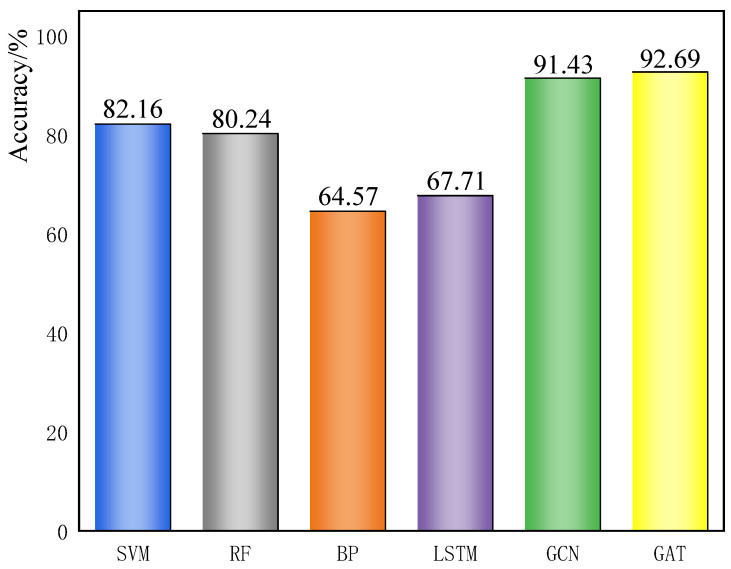
Different model test cases.

When the sample size was insufficient, we tested different models separately. From the 2080 samples processed previously, 520 samples were taken as the training set, 75 samples as the validation set, and 148 samples as the test set, keeping the main hyperparameters consistent. The datasets were then trained and tested with different models, and the specific test results are shown in Figure 12.

As can be seen in Figure 12, the graph neural network still has a good localization effect in the case of a small number of data samples, and its test results are significantly better than those of the traditional BP and LSTM networks. Like many other neural networks, the BP and LSTM networks need a large number of training samples to train the weight parameters of the model, while GNN networks can learn the information of the whole data structure from the connections between the whole data samples, which reduces the learning cost. SVM and RF diagnostic methods also have good diagnostic results when dealing with small-sample data, which is the advantage of their algorithmic structures. However, they still have shortcomings when compared with the GCN and GAT networks. The results of GAT are slightly better than GCN. In terms of the overall results, graph neural networks outperform the other methods in the case of small sample sizes, and the learning sample cost required is lower than that of traditional machine learning and deep learning methods.

## 8. Model Robustness Testing

In the actual fault diagnosis, there is a loss or distortion of the fault characteristic information of the secondary equipment, which results in the wrong expression of the characteristic information. Finally, the wrong expression can influence the final diagnosis results. For example, the abnormal sampling of protection equipment is often used as an important feature to diagnose problems occurring in relay protection sampling circuits. Once the information sent by this device is incorrect, it greatly increases the difficulty of detection. In the case of a line merging unit-switch port failure, for example, when this failure occurs, the merging unit issues a self-test alarm after discovering a device abnormality through self-test. In addition, the merging unit, in turn, issues a total GOOSE alarm because the GOOSE data are abnormal, which results from the failure to receive the relevant message information. The measurement and control unit also fails to receive the relevant message information. The collected characteristic information is obtained using Equation (20).

(20) $$\{\begin{array}{l} \{\begin{array}{l} X_{A}=\left\{X_{Mi},X_{Pi},X_{ITi}\right\}\\ X_{Mi}=\left\{0,0,1,1,1,\ldots ,1,0,1,\ldots \right\}\\ X_{Pi}=\left\{0,0,\ldots ,0\right\}\\ X_{ITi}=\left\{0,0,\ldots ,0\right\} \end{array}\\ X_{B}=\left\{0,0,1,0,0,1,\ldots ,1,\ldots \right\}\\ \begin{array}{l} X_{C}=\{3.014,3.011,3.016,\\ \;57.437,57.453,57.429,\\ \;3.009,3.022,3.015,\\ \;57.433,57.435,57.431\} \end{array} \end{array}$$

If the protection device wrongly sends an abnormal message of sampling data, then $X_{Pi}$ in Equation (20) changes to $X_{pi}^{\prime }=\left\{0,0,\ldots ,1,\ldots \right\}$. Substituting them into the previous six different models for fault localization, the results are obtained as shown in Equation (21).

(21) $$\{\begin{array}{l} \mathrm{SVM}=[0,\ldots ,\mathrm{NO}.11,\ldots ,\mathrm{NO}.16,\ldots ,0]\\ \mathrm{RF}=[0,\ldots ,\mathrm{NO}.11,\ldots ,\mathrm{NO}.16,\ldots ,0]\\ \mathrm{BP}=[0,\ldots ,\mathrm{NO}.11,\ldots ,0]\\ \mathrm{LSTM}=[0,\ldots ,\mathrm{NO}.11,\ldots ,0]\\ \mathrm{GCN}=[0,\ldots ,\mathrm{NO}.11,\ldots ,0]\\ \mathrm{GAT}=[0,\ldots ,\mathrm{NO}.11,\ldots ,0] \end{array}$$

From Equation (21), it can be seen that, in the case of the erroneous protection unit sampling information, SVM and RF incorrectly predicted the protection unit SV board failure (No. 16) in addition to the merging unit-switch port failure (NO.11). Whereas, the BP, LSTM, GCN, and GAT models made correct judgments.

Under these conditions, the protection device wrongly issued the sampling data abnormality message and, for some reason, caused the protection device to issue the SV total alarm. $X_{Pi}$ in Equation (20) will change to $X_{pi}^{\prime \prime }=\left\{0,0,\ldots ,1,\ldots ,1\ldots \right\}$. The results obtained after substituting them into different diagnostic models, respectively, are shown in Equation (22).

(22) $$\{\begin{array}{l} \mathrm{SVM}=[0,\ldots ,\mathrm{NO}.11,\ldots ,\mathrm{NO}.16,\ldots ,\mathrm{NO}.19,\ldots ,0]\\ \mathrm{RF}=[0,\ldots ,\mathrm{NO}.11,\ldots ,\mathrm{NO}.16,\ldots ,\mathrm{NO}.19,\ldots ,0]\\ \mathrm{BP}=[0,\ldots ,\mathrm{NO}.11,\ldots ,\mathrm{NO}.19,\ldots ,0]\\ \mathrm{LSTM}=[0,\ldots ,\mathrm{NO}.11,\ldots ,0]\\ \mathrm{GCN}=[0,\ldots ,\mathrm{NO}.11,\ldots ,0]\\ \mathrm{GAT}=[0,\ldots ,\mathrm{NO}.11,\ldots ,0] \end{array}$$

From Equation (22), it can be seen that, when the above fault characteristic information distortion occurs, the fault types that SVM and RF predicted incorrectly are No. 16 (protection device SV board fault) and No. 19 (merging unit-protection device port fault). The faults misjudged by the BP network are also related to No. 16. The LSTM, GCN, and GAT models all made correct judgments.

In order to examine the immunity of different models to interference across the border, 100 samples were selected as interference information input in the test set. First, the expression of the feature information in these samples was artificially changed. For example, the information with the feature “0” was changed to “1” and the information with the feature “1” was changed to “0”. The number of changed feature information was limited to 2. After that, the error tolerance was analyzed by simulating the abnormal information situation with different methods as mentioned above, and Table 9 shows the results of different methods for testing the interference dataset.

As can be seen in Table 9, the SVM method is the most affected under the test with the interference dataset, while GAT has a smaller decrease in accuracy and better fault tolerance performance compared to the other five methods.

## 9. Conclusions

Regarding the problems of traditional methods with low efficiency, artificial intelligence methods with a high cost of samples and poor portability, and frequent upgrades of models, this study proposes a fault diagnosis method based on a graph attention network. First, the expression of the features is proposed by combining the feature information exhibited by the corresponding detection nodes at the time of a fault’s occurrence, and the feature set is constructed into the form of a graph data structure based on the KNN algorithm. Then, a fault diagnosis model is established based on GAT, and the proposed method is validated by taking multi-interval faults of a 220kV intelligent substation as an example. Finally, the advantages and disadvantages of different methods under different conditions are compared and analyzed.

By comparing the graph attention network with traditional machine learning and deep learning, the advantages of GAT are as follows:1. Higher fault localization accuracy; 2. Faster model training; 3. Better capability of dealing with dynamic graph problems; 4. Better localization results under the condition of small sample sizes; 5. Better robustness. For model updating, the proposed KNN-based graph structure construction method can automatically construct graph data when new faults are added. When new fault feature information is added to the existing graph structure, it automatically finds the structural relationship between the fault information and generates the graph structure required for GAT, which reduces the difficulty of model updating. It provides a new idea and method for the operation and maintenance of intelligent substations.

## Figures and Tables

**Table 1:** Diagnosed object.

Interval Name	Symbol Abbreviations	Symbol Meaning
Bus interval	MU_1_	Bus merger unit
	P_1_	Bus protection device
	IT_1_	Bus intelligent terminal
	M&C_1_	Bus measurement and control device
Line interval	MU_2_	Line merger unit
	P_2_	Line protection device
	IT_2_	Line intelligent terminal
	M&C_2_	Line measurement and control device
Transformer interval	High-voltage side MU_3_	Transformer high-voltage side merger unit
	Low-voltage side MU_4_	Transformer low-voltage side merger unit
	P_3_	Transformer protection device
	Ontology IT_3_	Transformer body intelligent terminal
	High-voltage side IT_4_	Transformer high-voltage side measurement and control device
	Low-voltage side IT_5_	Transformer low-voltage side measurement and control device
	M&C_3_	Transformer measurement and control device

**Table 2:** Interval information flow.

No.	Sending Port	Receiving Port	Sending Method
1	MU_1_	M&C_1_	Networking SV
2	MU_1_	P_1_	Point-to-Point SV
3	IT_1_	P_1_	Point-to-Point GOOSE
4	IT_1_	MU_1_	Networking GOOSE
5	IT_1_	M&C_1_	Networking GOOSE
6	P_1_	IT_1_	Point-to-Point GOOSE
7	M&C_1_	MU_1_	Networking GOOSE
8	M&C_1_	IT_1_	Networking GOOSE
9	MU_2_	P_1_	Point-to-Point SV
10	P_1_	IT_2_	Point-to-Point GOOSE
11	IT_2_	P_1_	Point-to-Point GOOSE
12	MU_1_	MU_2_	Networking GOOSE
…	…	…	…
47	P_1_	IT_5_	Point-to-Point GOOSE
48	P_1_	P_3_	Networking GOOSE

**Table 3:** Fault type.

Type Number	Fault Type
00000	Merge unit sampling exception
00001	Merge unit misconfiguration
00010	Consolidation unit communication failure
00011	Protection device sampling communication failure
00100	Protection device GOOSE board failure
00101	Tester power failure
⋮	⋮
11100	Communication failure
11101	Packet loss in communication messages

**Table 4:** Network parameters setting.

Parameters	Value
Initial learning rate	0.005
Decay of weights	0.0005
Feat_dropout	0.1
Attn_dropout	0.1
Batch_size	Full
Number of iterations	2000
Optimizer	Ranger

**Table 5:** Model test situation.

Network Layer	Attention Head	Accuracy (%)
GAT layer = 1	Head = 6	97.64%
GAT layer = 1	Head = 8	98.24%
GAT layer = 1	Head = 10	99.08%
GAT layer = 1	Head = 12	96.73%
GAT layer = 2	Head = 6	98.63%
GAT layer = 2	Head = 8	98.74%
GAT layer = 2	Head = 10	99.45%
GAT layer = 2	Head = 12	91.92%
GAT layer = 3	Head = 6	97.69%
GAT layer = 3	Head = 8	97.92%
GAT layer = 3	Head = 10	97.12%
GAT layer = 3	Head = 12	90.38%

**Table 6:** Specific discriminatory situations.

Fault Number	Sample Size	Number of SVM Errors	Number of RF Errors	Number of GAT Errors
1	24	2	2	0
5	24	0	9	0
13	24	7	9	0
14	24	0	5	0
15	24	0	5	0
18	24	7	12	1
20	24	10	12	0
23	24	7	12	1
24	24	13	15	2
26	24	7	6	1
other	384	0	0	0

**Table 7:** Comparison of iteration times.

Method	GAT	GCN	LSTM	BP
T1	76.297 s	52.610 s	150.111 s	37.749 s
T2	42.508 s	45.827 s	244.025 s	11.005 s

**Table 8:** Training set and test set K value settings.

Group Number	K Value Setting Situation	K Value Setting Situation	K Value Setting Situation
Group A	Training set k = 2, test set k = 2 (D1 in Figure 11)	Training set k = 2, test set k = 3 (D2 in Figure 11)	Training set k = 2, test set k = 4 (D3 in Figure 11)
Group B	Training set k = 3, test set k = 2 (D4 in Figure 11)	Training set k = 3, test set k = 3 (D5 in Figure 11)	Training set k = 3, test set k = 4 (D6 in Figure 11)
Group C	Training set k = 4, test set k = 2 (D7 in Figure 11)	Training set k = 4, test set k = 3 (D8 in Figure 11)	Training set k = 4, test set k = 4 (D9 in Figure 11)

**Table 9:** Interference dataset testing.

Model	Interference-Free Dataset	Interference Dataset	Change in Accuracy
SVM	88.43%	84.22%	−4.21%
RF	86.24%	83.05%	−3.19%
BP	90.23%	89.20%	−1.03%
LSTM	98.25%	97.62%	−0.63%
GCN	98.54%	98.43%	−0.11%
GAT	99.02%	98.93%	−0.09%

## Data Availability

Data are contained within the article.

## References

[1] Kriger C., Behardien S., Retonda-Modiya J. (2013). A Detailed Analysis of the GOOSE Message Structure in an IEC 61850 Standard-Based Substation Automation System. Int. J. Comput. Commun. Control.

[2] Dong X., Wang D., Zhao M., Wang B., Shi S., Apostolov A. (2016). Smart power substation development in China. CSEE J. Power Energy Syst..

[3] Liu Y., Gao H., Gao W., Peng F. (2017). Development of a Substation-Area Backup Protective Relay for Smart Substation. IEEE Trans. Smart Grid.

[4] Law C.T., Bhattarai K., Yu D.C. (2008). Fiber-optics-based fault detection in power systems. IEEE Trans. Power Del..

[5] Ye Y., Sun Y., Huang T., Guo M., Huang Y. (2016). Online state detection and fault diagnosis technology of relay protection secondary circuits in smart substation. Power Syst. Prot. Control.

[6] Wang T., Xie M., Sun Y., Shen P. (2015). Analysis of reliability for relay protection systems in smart substation. Power Syst. Prot. Control.

[7] Wang H., Xu H., Tong X., Diao X., Guo S., Zheng Y. (2019). The online disturbance degree assessment method of protection systems in intelligent substation based on the structure entropy weight method and fault trees. Power Syst. Technol..

[8] Dai Z., Lu H., Liu Y., Liu B., Chen Y. (2019). Fault Diagnosis Method of secondary Circuit of Intelligent Station Protection Based on Improved D-S Evidence Theory. Power Syst. Prot. Control.

[9] Shi Z., Yao W., Li Z., Zeng L., Zhao Y., Zhang R., Tang Y., Wen J. (2020). Artificial intelligence techniques for stability analysis and control in smart grids: Methodologies, applications, challenges and future directions. Appl. Energy.

[10] Ren B., Li J., Zheng Y., Chen X., Zhao Y., Zhang H., Zheng C. (2020). Research on Fault Location of Process-Level Communication Networks in Smart Substation Based on Deep Neural Networks. IEEE Access.

[11] Ren B., Zheng Y.K., Wang Y.F., Sheng Q., Li J., Zhang H., Zheng C. (2021). Research on fault localization of intelligent substation secondary equipment based on deep learning. Grid Technol..

[12] Chen G., Dong X., Zheng Y., Xu H. (2022). Research on Fault Diagnosis of Relay Protection Test Based on Long and Short Term Memory Network. Power Syst. Prot. Control.

[13] Zhang C., Zheng Y., Lu J., Zhang H., Ren H., Yang Z. (2022). Research on secondary circuit fault localization of intelligent substation based on graph neural network. Power Syst. Prot. Control.

[14] Cheng X., Lee W.J., Pan X. (2017). Modernizing Substation Automation Systems: Adopting IEC Standard 61850 for Modeling and Communication. IEEE Ind. Appl. Mag..

[15] Shin S., Yang H. (2023). Performance Analysis of Routable GOOSE Security Algorithm for Substation Communication through Public Internet Network. Sensors.

[16] Lozano J.C., Koneru K., Ortiz N., Cardenas A.A. (2023). Digital Substations and IEC 61850: A Primer. IEEE Commun. Mag..

[17] Yang Q., Hao W., Ge L., Ruan W., Chi F. (2019). Farima model-based communication traffic anomaly detection in intelligent electric power sub-stations. IET Cyber-Phys. Syst. Theory Appl..

[18] Gururajapathy S.S., Mokhlis H., Illias H.A. (2017). Fault location and detection techniques in power distribution systems with distributed gen-eration: A review. Renew. Sustain. Energy Rev..

[19] Zhang Y., Cai Z., Long F., Li X., Su Z. (2016). Real-time fault diagnosis model and method for intelligent substation communication network. Grid Technol..

[20] Liu W., Wang H., Zhang Y. (2014). Intelligent substation process layer network message characteristics analysis and communication configuration research. Power Syst. Prot. Control.

[21] Yang T., Zhao R., Zhang W., Yang Q. (2017). On the modeling and analysis of communication traffic in intelligent electric power substations. IEEE Trans. Power Del..

[22] Yaniv A., Kumar P., Beck Y. (2023). Towards adoption of GNNs for power flow applications in distribution systems. Electr. Power Syst. Res..

[23] He L., Bai L., Yang X., Du H., Liang J. (2023). High-order graph attention network. Inf. Sci..

[24] Liang F., Qian C., Yu W., Griffith D., Golmie N. (2022). Survey of Graph Neural Networks and Applications. Wirel. Commun. Mob. Comput..

[25] Liao W., Bak-Jensen B., Pillai J.R., Wang Y., Wang Y. (2022). A Review of Graph Neural Networks and Their Applications in Power Systems. J. Mod. Power Syst. Clean Energy.

[26] Li T., Zhou Z., Li S., Sun C., Yan R., Chen X. (2022). The emerging graph neural networks for intelligent fault diagnostics and prognostics: A guideline and a benchmark study. Mech. Syst. Signal Process..

[27] Liang J., Tang J., Gao F., Wang Z., Huang H. (2023). On region-level travel demand forecasting using multi-task adaptive graph attention network. Inf. Sci..

[28] Li J., Wang X., He J., Zhang Y., Zhang D. (2021). Distribution Network fault location Method based on Graph Attention Network. Power Grid Technol..

[29] Ye Y., Sun Y., Huang T., Guo M., Huang Y. (2016). Intelligent substation relay protection secondary circuit online Monitoring and fault diagnosis technology. Power Syst. Prot. Control.

